# Quantum dot enhanced photonic biosensors for single cell analysis from synthesis to clinical application

**DOI:** 10.1186/s11671-026-04483-z

**Published:** 2026-02-24

**Authors:** Mohammad Hosseini Hooshiar, Javad Hosseini Hooshiar, Saeed Hosseini Hooshiar

**Affiliations:** 1 Department of Periodontology, School of Dentistry, Tehran University of Medical Sciences, Tehran, Iran; 2 Sabzevar University of Medical Sciences, Tehran, Iran

**Keywords:** Quantum dots, Biosensors, Fluorescence, Nanoparticles, Biomarkers, Cancer, Diagnostics

## Abstract

The quantum dot (QD)-enhanced optical biosensors are very sensitive tools for analyzing single cells. Heavy metal-free alternatives, such as indium phosphide and carbon-based QDs, address safety concerns while achieving high quantum efficiency. Photonic enhancement techniques provide 240-fold signal amplification and quality factors of up to 7000, allowing femtomolar detection limits. However, clinical translation encounters manufacturing scalability and stability problems in complex biological matrices. This review discusses synthetic techniques, surface functionalization, and clinical implementation, highlighting important challenges and potential for improving QD-based biosensors.

## Introduction

Quantum dot (QD) sensors reach detection limits as low as ~ 1 copy/mL for the influenza virus H1N1 RNA and 0.041 U/mL for the activity of tyrosinase, which is an enormous sensitivity improvement when compared against the existing CdSe/ZnS-based probes, unable to detect extremely low viral concentrations [1, 2]. Clinical evidence demonstrates QD immunoassays achieving 92.3–98.1% positive rates in comparison with 78.3–83.1% for the conventional ELISA protocols, with response times reduced to 5 min [3]. Scalability in production is made difficult by 40–60% rejection rates for pharmacopeia-grade production with high-quality specifications [4].

Single-molecule sensitivity enables circulating tumor cell detection with limits as low as 3 cells/mL, demonstrating linear dynamic ranges spanning 300 cells/mL to 6 × 10^5^ cells/mL in complex serum matrices with coefficients of determination (R^2^) exceeding 0.995. This is higher than for conventional techniques that need to operate at much higher concentrations [5]. CdSe/ZnS core–shell QDs can attain quantum yields of 85–95% in the laboratory. They can narrow emission linewidths to as low as 20–35 nm FWHM. Gradient-shell designs can reach 98% quantum efficiency [6, 7]. Performance, however, drops from laboratory values of 85–95% to 50–80% in biological surroundings due to the formation of the protein corona and conditions of the surroundings [8]. Time-resolved measurements show energy transfer efficiencies as high as 71.5% with lifetime changes from 5.45 to 1.51 ns [9–12].

Photoluminescence quantum yields of indium phosphide QDs reach 91%, but the QDs present negligible cytotoxicity levels of up to 1000 μg/mL, mitigating the issue of safety [13]. Carbon QDs reach particle diameters of 1.30 to 11.40 nm with quantum yield values of 1 to 50% based on synthesis conditions, although batch-to-batch variations hinder standardization [14, 15]. Detection limits as low as 0.041 U/mL for the analysis of the enzyme were observed for silicon QD systems, accompanied by high biocompatibility [2, 16–18]. Integrating with photonics offers fluorescence enhancement factors of more than 240-fold via plasmonic-photonic combined effects [19]. Bound states in the continuum obtain quality factors of 7000 with photoluminescence enhancement of eight-fold, although the sheer complexity of manufacture restricts applicability [19]. Also, QD heterogeneous integration in silicon nitride waveguides obtains external quantum efficiencies of 67.5% at 1275 nm [20–22]. Some of the applications with success involve the detection of Trichinella spiralis with 100% selectivity against 90% for the commercial ELISA tests, with the response time lowered to 25 min [23]. Multiplexed lateral flow tests attain detection limits of 56, 120, and 41 copies/mL for SARS-CoV-2, adenovirus, and influenza A, respectively, with the result provided in 20 min [24]. This review is the first comprehensive analysis integrating QD synthesis advances, photonic enhancement mechanisms, and clinical translation barriers in a unified framework.

## QD fundamentals and photophysical properties

**Fig. 1:**
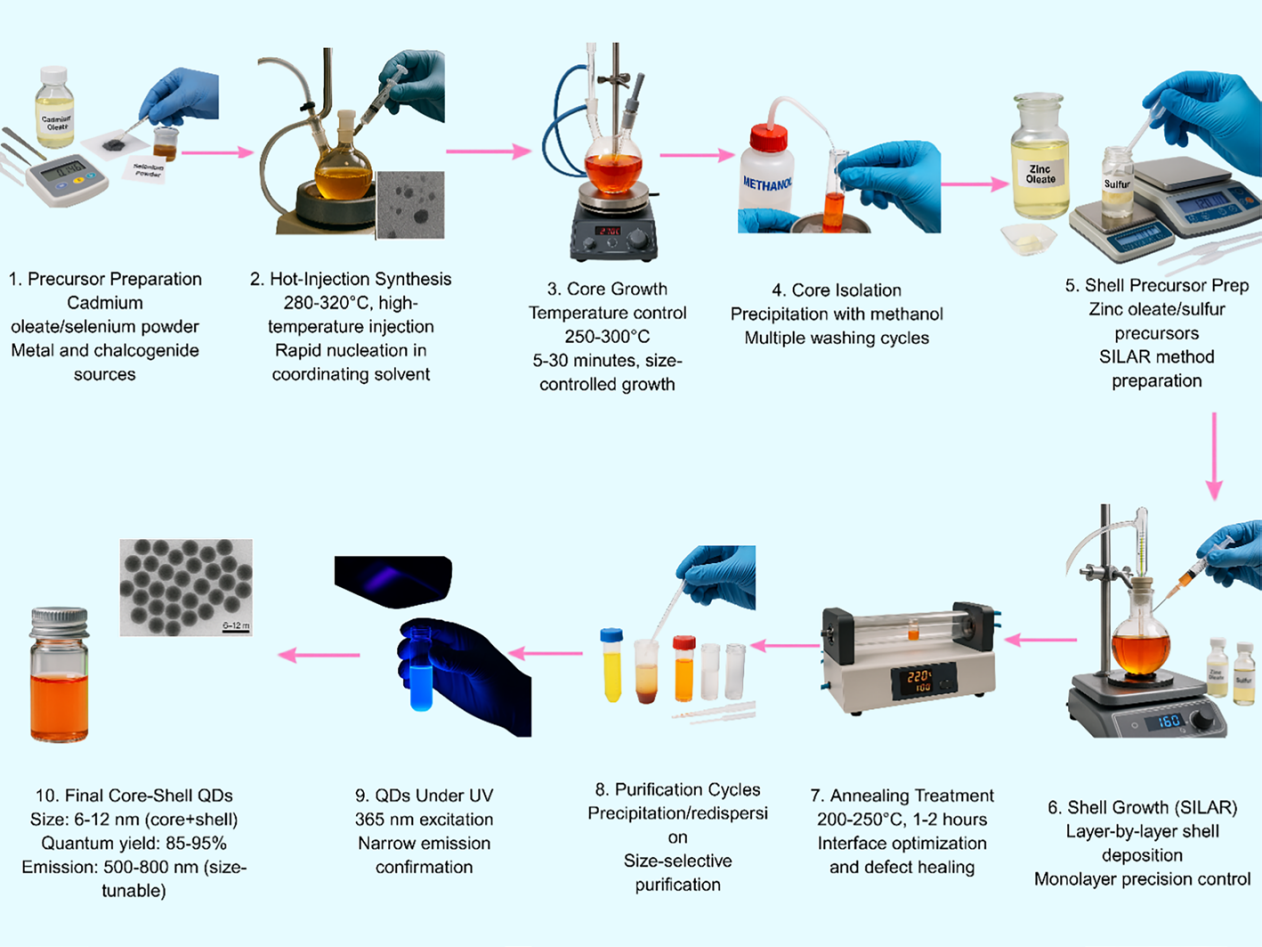
Synthesis workflow of CdSe/ZnS QDs via hot-injection and SILAR methods

Quantum dots (QDs) have characteristic optical behavior regulated by the quantum confinement effect, when the size of semiconductor nanocrystals is in the range of or smaller than the exciton Bohr radius of the material. QDs, in terms of the composition of the semiconductor material, generally vary in size from about 1 to 10 nm, allowing for sharp control of the optical properties [25]. Figure 1 illustrates the complete synthesis workflow of CdSe/ZnS core–shell QDs via hot-injection and SILAR methods.

### Advanced core–shell architectures: summary and assessment

**Fig. 2:**
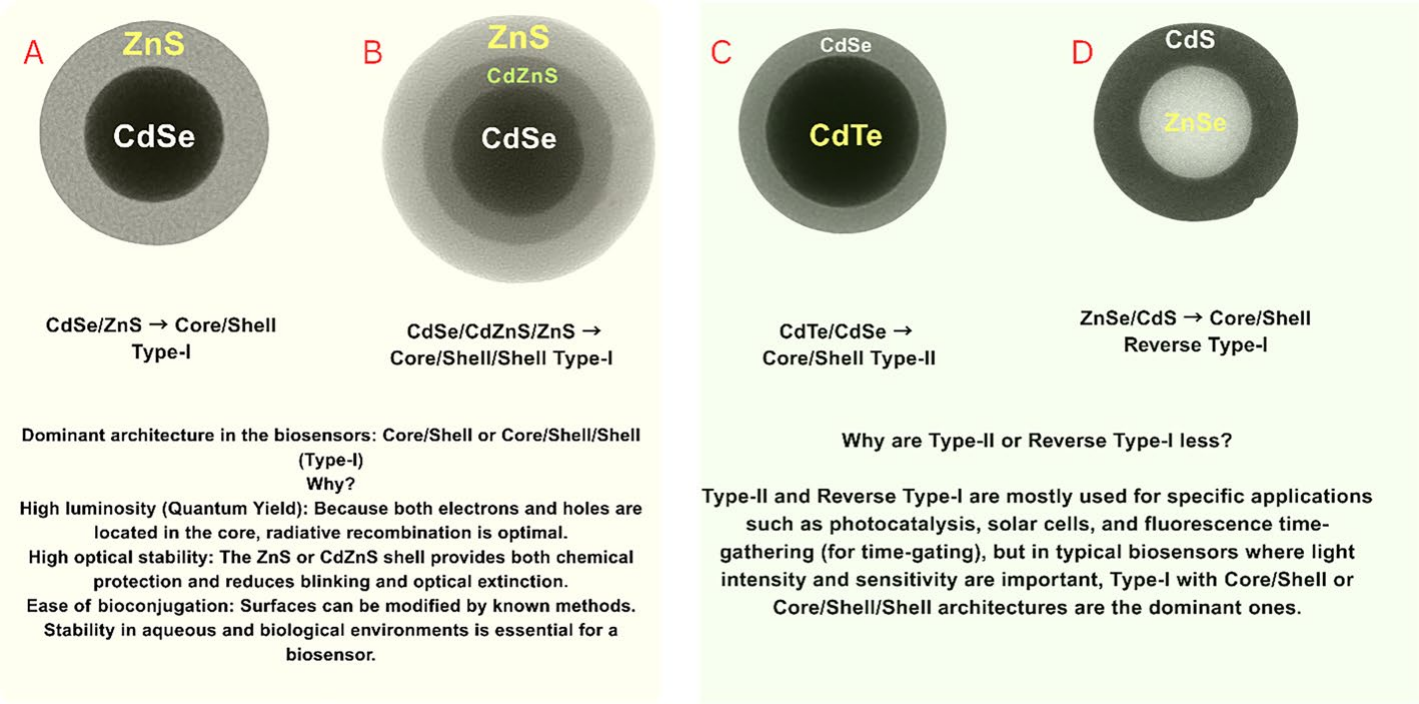
Schematic representation of core–shell QD architectures with different band alignments. **A** Type-I CdSe/ZnS core/shell system with high quantum yield due to optimal radiative recombination. **B** Type-I CdSe/CdZnS/ZnS core/shell/shell architecture with enhanced optical stability. **C** Type-II CdTe/CdSe heterostructure with extended radiative lifetimes. **D** Reverse Type-I ZnSe/CdS architecture

Type-I core–shell configurations like CdSe/ZnS attain high quantum yields of 85 to 95% with narrow emission linewidths of 20–35 nm FWHM. Sophisticated gradient-shell structures attain even higher quantum efficiency, as high as 98%, optimally in the lab (Fig. 2) [7, 26–28].

QDs face interfacial challenges in biological environments that compromise biosensor performance through protein corona formation, where biological fluid proteins spontaneously adsorb onto surfaces, altering hydrodynamic properties, surface charge, and optical characteristics. The biggest challenge for core–shell quantum dots (QDs) is the loss of performance when moving from well-controlled laboratory settings to complicated biological media. Placing quantum dots (QDs) in biological fluids can lower their quantum yield by 30–70% [29]. Environmental factors, like the fluctuations of pH, changes in ionic strength, and the concentration of metal ions, also impact QD steadiness and fluorophore behavior. These factors affect the surface charge, ligand protonation states, as well as bring about mechanisms of fluorescence quenching, in turn leading to detection limits of as low as 0.1 nM for the mercury-induced quenching, as well as nanomolar sensitivity for the interfingent transition metal susceptibility [8, 30, 31].

## Next-generation core–shell architectures

### Type I and advanced shell designs

Type-I core–shell constructs, like CdSe/ZnS quantum dots (QDs), attain high quantum yields of 85 to 95%, with narrow emission linewidths of 20–35 nm full-width half-maximum (FWHM). This is a much higher performance than is typically attainable in classic organic fluorophores. Besides, sophisticated gradient-shell configurations have been reported to attain quantum efficiencies of nearly 98% in specially optimized laboratory settings [7, 26]. New-generation QD systems present brightness values roughly in the range of 10–100-fold higher when compared to classic fluorophoric dyes, along with superior photostability, sustaining the fluorescence signal 7–eightfold longer when continually irradiated [27, 28]. Table 1 compares different QD types, summarizing their optical properties, biocompatibility, and key advantages and limitations for biosensing applications.

**Table 1:** Comparison of different QD types for biosensing applications

QD Type	Quantum yield	Emission linewidth	Toxicity	Stability in biological media	Key advantages	Key limitations
CdSe/ZnS (Type-I)	Very high	Narrow	High (heavy metal)	Moderate	Highest brightness, narrow emission, well-established synthesis	Heavy metal toxicity, regulatory concerns, and performance degradation in biological fluids
InP/ZnSe/ZnS	High	Moderate	Low	Good	Heavy metal-free, high biocompatibility, suitable for clinical translation	Broader emission than CdSe, complex synthesis optimization
Carbon QDs	Low to moderate	Variable	Very low	Excellent	Green synthesis, excellent biocompatibility, water soluble	Lower quantum yield, batch-to-batch variability, broader size distribution
Silicon QDs	Moderate	Tunable	Very low	Good	High biocompatibility, no aggregation-caused quenching, safe for clinical use	Lower brightness than cadmium-based QDs
Perovskite (CsPbX_3_)	Very high	Narrow	Moderate (lead content)	Poor	High brightness, tunable emission, defect-tolerant	Instability in aqueous environments, lead toxicity concerns, and limited clinical applicability

### Gradient-shell architectures and performance optimization

Gradient-shell quantum dots have a gradual change in composition from the core to the outside. This structure improves lattice matching and reduces strain at the interface. As a result, there are fewer defects and less nonradiative energy loss [1, 32]. Performance optimization, using precision in shell composition gradient control, interface engineering, heat treatment regimens, and advanced surface passivation, achieved quantum efficiencies of as much as 98 percent as well as 20- to 35-nm emission linewidths. Transmission electron microscopy (TEM) typically shows 3.5–5-nm-sized core diameters, along with shell thicknesses of about 2- to 3-nm, for these high-performance systems [33, 34].

### Type-II and quasi-type-II band alignments

Band alignment engineering in quantum dots (QDs) has enabled the design of type-II and quasi-type-II heterostructures, where electrons and holes are deliberately separated into different regions across the core–shell interface. In type-II QDs, for example, the electron typically localizes in the shell while the hole remains confined within the core (or vice versa), whereas quasi-type-II structures feature partial separation depending on material combinations and band offsets. This spatial separation significantly reduces electron–hole recombination rates, resulting in radiative lifetimes that can exceed 100 ns, which is substantially longer than those observed in type-I QDs. Type-II and quasi-type-II architectures demonstrate extended radiative lifetimes (> 100 ns) that enable time-gated fluorescence detection, providing temporal discrimination against sub-nanosecond biological autofluorescence and enhancing signal-to-noise ratios by 5–tenfold in complex biological matrices.

### Heavy metal-free QD alternatives

#### Indium phosphide (InP) systems

Heavy metal-free cadmium-based QD alternatives, indium phosphide (InP) quantum dots, are the preferred substitute for biomedical applications from the viewpoint of safety as well as regulations [35]. Indium phosphide (InP) quantum dots offer superior performance in optical applications due to the InP/ZnSe/ZnS core–shell configurations. They exhibit up to 91% photoluminescence quantum yield in the best laboratory conditions. Some of the systems attain 87% quantum yield with sharp emission linewidths of about 48 nm FWHM. This renders them well-suited for applications in biosensing, for applications of high biocompatibility, as well as high sensitivity [13]. Sophisticated strategies of surface engineering applied in the synthesis of InP QDs efficiently obviated past synthesis challenges, specifically via selenium shielding coatings that inhibit cation exchange reactions, as well as quasi-shell synthesis strategies that optimize optical performance as well as biological stability [13, 35]. Clinical translation prospects for InP-based biosensors are up-and-coming due to their demonstrated effectiveness in practical applications, including bacterial detection through aptamer-conjugated systems for specific pathogen labeling, food safety monitoring via fluorescence-visualized sensors for fish freshness assessment, and biomedical imaging applications using highly stable biotemplated QD systems for in situ bacterial monitoring [36–39].

#### Carbon QDs and green synthesis

**Fig. 3:**
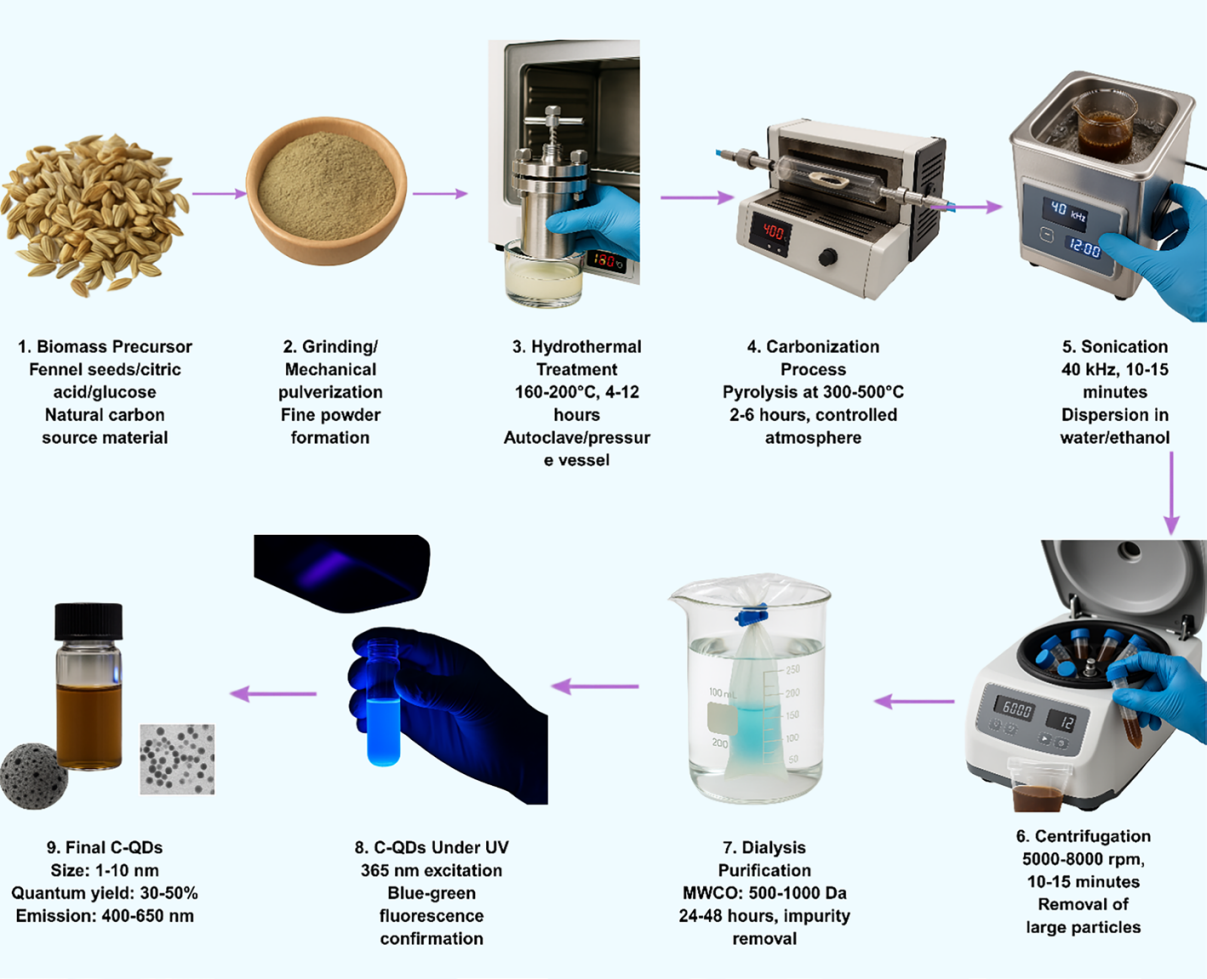
Green synthesis workflow of carbon QDs from biomass precursors through hydrothermal treatment, carbonization, and purification steps

Carbon QDs are alternatives to traditional semiconductor QDs, demonstrating outstanding biocompatibility with negligible cytotoxicity up to concentrations of 1000 μg/mL and excellent water solubility, colloidal stability, and resistance to photobleaching (Fig. 3) [40, 41]. The synthesis from natural sources has demonstrated versatility, with carbon dots derived from materials including pineapple leaves, orange peel, mussels, tamarind shell powder, and medicinal plant extracts, achieving particle sizes ranging from 1.30 to 11.40 nm and quantum yields between 1 and 50% depending on synthesis conditions and precursor materials [14, 42]. The fluorescence properties of carbon dots are highly dependent on synthesis parameters, including temperature, pH, precursor type, and reaction time, leading to batch-to-batch variability that complicates standardization and reproducibility for commercial applications [43]. Surface functionalization remains challenging, as carbon dots often require complex modification procedures to achieve specific targeting capabilities, and the heterogeneous nature of carbon dot surfaces can lead to inconsistent bioconjugation efficiency [41]. Additionally, while green synthesis approaches are environmentally favorable, they often result in broader size distributions and less controlled optical properties compared to conventional synthesis methods, requiring purification and characterization procedures to ensure consistent performance [44–46].

#### Silicon QDs and biocompatible alternatives

**Fig. 4:**
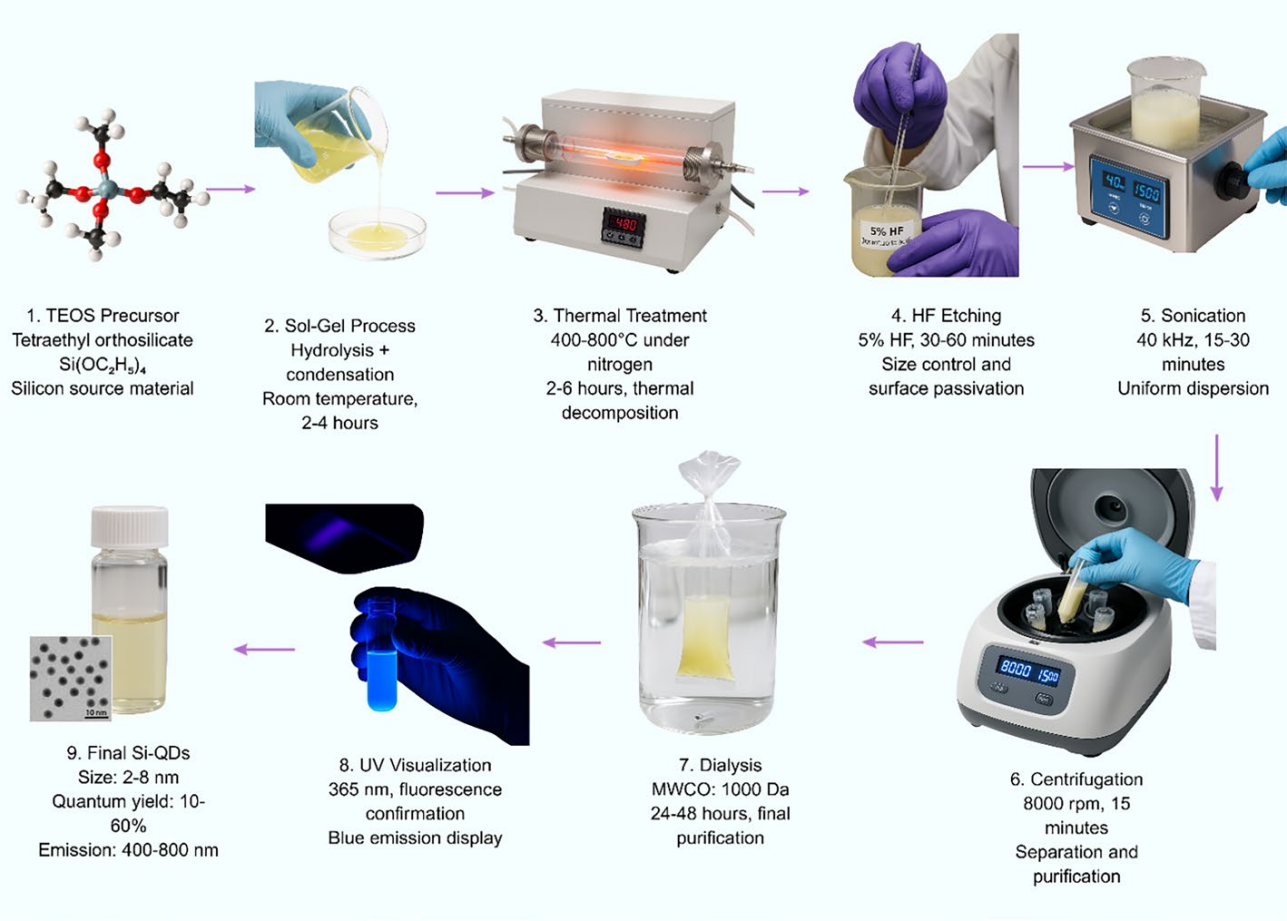
Sol–gel synthesis workflow of silicon QDs using TEOS precursor through hydrolysis, thermal treatment, HF etching, and purification, producing biocompatible QDs with tunable emission properties

Silicon quantum dots are drawing attention for biosensing because they produce bright light, dissolve well in water, and remain stable during use. These features make it easy to attach different molecules to their surface for specific detection tasks. Unlike some other quantum dots, silicon QDs do not clump together or lose brightness, so they avoid problems with reduced signal known as aggregation-caused quenching (Fig. 4) [47]. Scientists can make silicon-doped carbon quantum dots with simple hydrothermal methods. These quantum dots show strong and steady fluorescence, which makes them useful for very sensitive biosensors. For example, researchers have used them to detect tyrosinase activity at very low concentrations, down to 0.041 units per milliliter [2]. One major advantage of silicon quantum dots is their biocompatibility, meaning they are safe to use in medical tests. They have been used in clinical research to detect exosomes for cancer monitoring [48] and to measure brain-derived neurotrophic factor for diagnosing Alzheimer’s disease [49]. Because silicon quantum dots do not have the safety risks of heavy metals, they are seen as strong candidates for the next generation of biosensors that can be safely used in clinical practice [49].

### Perovskite QDs: emerging high-performance systems

#### Halide perovskite architectures

**Fig. 5:**
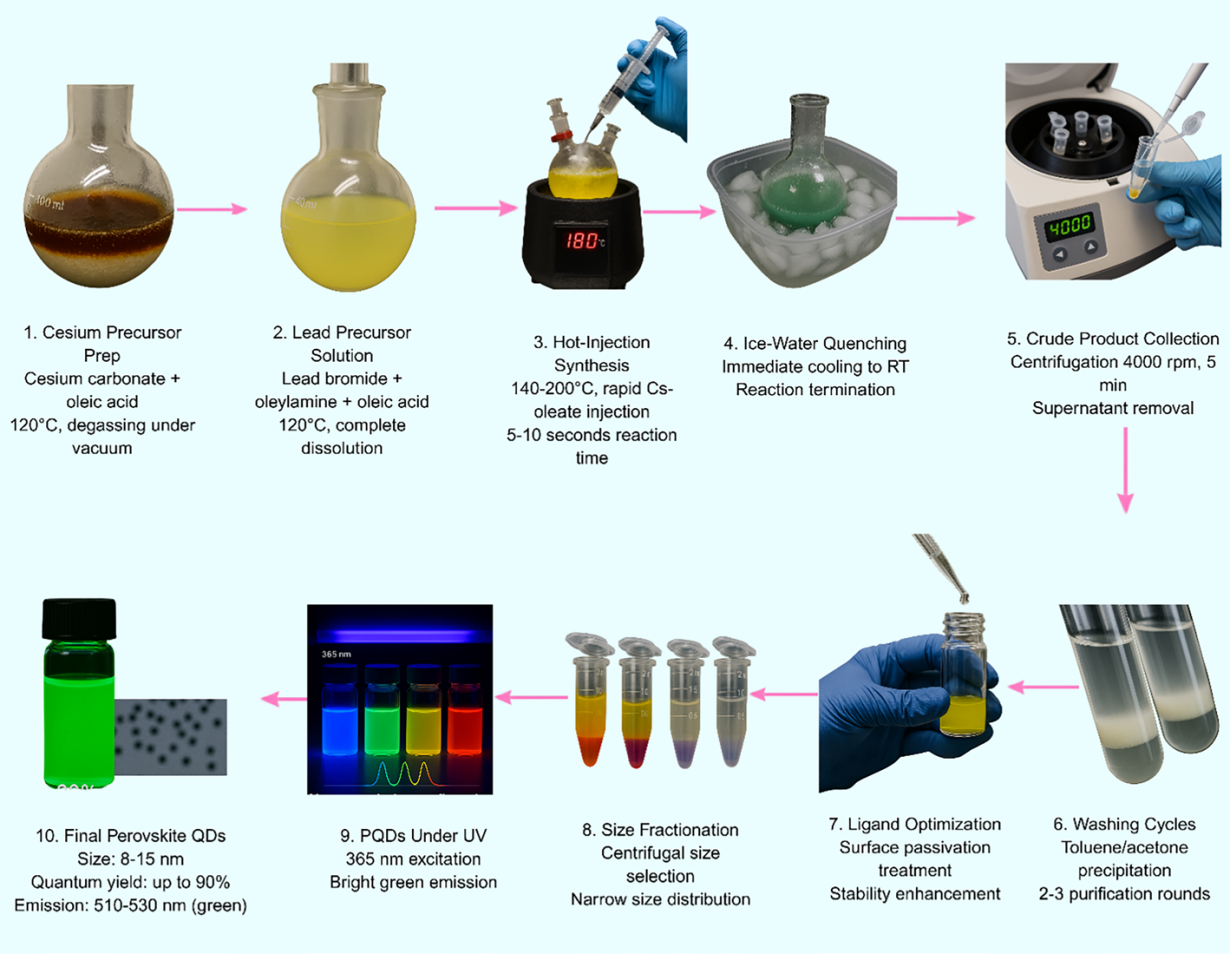
Hot-injection synthesis workflow of cesium lead halide perovskite QDs, achieving a quantum yield up to 90% and bright green emission through optimized temperature control and surface passivation

Cesium lead halide quantum dots (CsPbX_3_) are a new group of nanomaterials gaining popularity in biosensing because they offer strong and tunable light emission [50]. Scientists can adjust their color by changing their chemical composition, and these quantum dots can be made very bright and efficient, with some achieving quantum yields up to 90% and narrow emission bands (Fig. 5) [51]. The defect-tolerant lattice structure of these materials supports high radiative efficiency, while hot-injection synthesis at 140–200 °C yields particles with low size-dispersion, making them ideally suited for multiplexed detection applications [52]. Some new designs, such as putting CsPbBr₃ quantum dots inside organometallic shells, make them more stable in water and boost their signal. This has led to very sensitive tests, like detecting proteins such as interleukin-17A at very low concentrations [53]. The versatility of halide perovskite architectures extends to diverse biosensing modalities, including fluorescence-based detection systems capable of rapid chloride ion sensing in biological systems with detection limits of 1.82 mM and response times of approximately 1 s, demonstrating excellent water and pH stability through innovative surface passivation strategies [54].

Despite all these advances, perovskite quantum dots still have a major weakness: they are not very stable in water or under long exposure to air and light. This limits their use in real clinical settings, where stability and reliability are crucial [55]. Scientists are developing new protective coatings and encapsulation methods, like combining the quantum dots with proteins or using special ligands, which have helped improve water stability and detection sensitivity [55]. Innovative surface engineering approaches have demonstrated significant progress, including bovine serum albumin-perovskite composites achieving enhanced water stability with detection limits of 28 μM for L-ascorbic acid. [56], and ligand-engineered hydrophilic systems utilizing zwitterionic ligands that enable almost 100% photoluminescence quantum yield with fast anion exchange capabilities for multicolor biosensing [57]. Advanced protection strategies involving organometallic compound encapsulation have successfully addressed water decomposition issues while enhancing photocurrent intensity for photoelectrochemical applications. [53]. However, even with these advances, long-term stability in biological samples remains a problem. Until this challenge is solved, the use of perovskite quantum dots in routine medical diagnostics will stay limited [55].

## Surface chemistry and bioconjugation: bridging nanomaterials and biology

Adapting QDs from organic synthesis environments to aqueous biological systems presents technological challenges. This directly impacts biosensor performance and clinical translation [58]. This transformation requires ligand exchange strategies that must simultaneously preserve the QDs’ optical properties while ensuring colloidal stability in complex biological media [59]. Advances in silver chalcogenide QDs demonstrate the importance of surface engineering modifications that maintain near-infrared emission and exceptionally low toxicity, rendering them suitable for biological applications [58]. However, significant challenges persist in maintaining optical performance during phase transfer, with quantum yield variations ranging dramatically from 2.80 to 38.1% depending on surface modification strategies [33]. The process typically involves replacing hydrophobic ligands such as trioctylphosphine oxide and oleic acid with hydrophilic alternatives that provide both water solubility and functional groups for bioconjugation [60]. Membrane-targeted QD-based BACE1 sensors have achieved detection limits as low as 0.079 μM while maintaining stability under acidic conditions and providing enhanced fluorescence brightness compared to conventional fluorophores [61]. The complexity of achieving reproducible surface functionalization across different QD batches represents a major bottleneck in translating laboratory successes to commercial products, with variations in ligand density and orientation significantly affecting biosensor performance [59].

Advanced ligand systems incorporate multidentate coordination and polymer-based architectures. These provide superior alternatives to traditional small-molecule ligands. However, they face challenges in maintaining consistent surface coverage and preventing aggregation in high-salt biological buffers [62]. Fluorescence resonance energy transfer systems demonstrate quenching efficiencies reaching 91.0% through optimized donor–acceptor pair combinations, though achieving such performance requires precise control of spectral overlap and distance parameters [63]. Carbon dots and graphene QDs synthesized through green synthesis methods show promise for biological applications due to their water dispersibility and functionalized surface structure, yet reproducibility across different synthesis batches remains problematic [60]. Dihydrolipoic acid derivatives and other cyclic ligand systems provide enhanced stability through intramolecular chelation mechanisms, though the complex synthesis routes and scalability issues limit their widespread adoption [59]. The development of self-powered sensing platforms demonstrates enhanced performance with current density retention rates of 87.37% over long-term operation, though maintaining such stability in complex biological matrices presents ongoing challenges [64].

Ion exchange resins and solid-phase methodologies provide superior control over ligand concentrations and reaction kinetics compared to conventional solution-based techniques, with response times optimized to approximately 17 min for rapid diagnostic applications [65]. However, the challenge of achieving uniform surface coverage across all QDs in a batch remains problematic, with the complexity of surface chemistry optimization requiring systematic investigation of multiple parameters, including pH, temperature, and ligand-to-QD ratios, to achieve reproducible performance [59]. The translation from laboratory-scale synthesis to clinical applications continues to face hurdles in cost-effectiveness, regulatory approval, and long-term stability validation in diverse biological environments [66].

### Bioconjugation methodologies with quantitative assessment

**Fig. 6:**
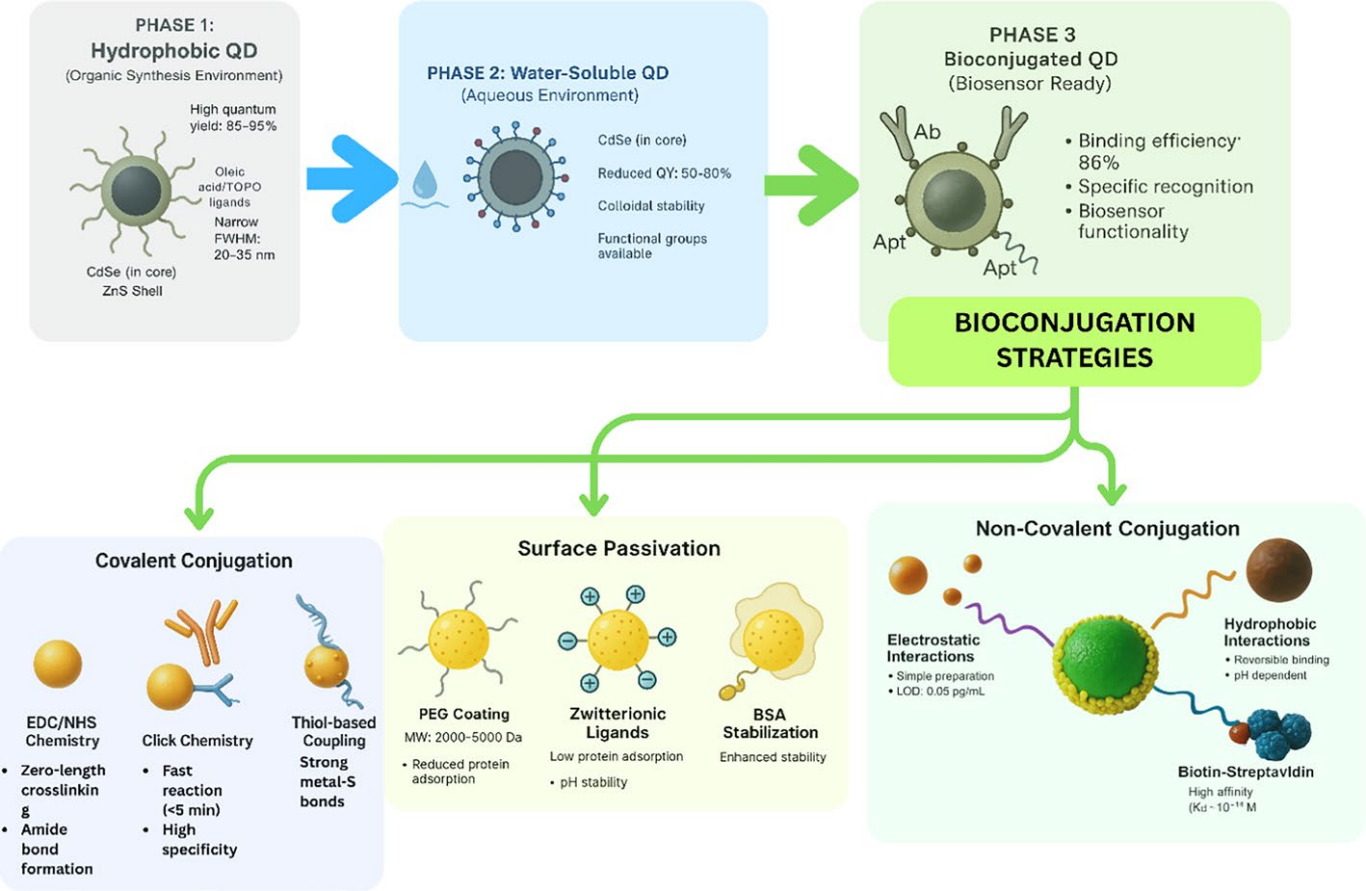
Surface chemistry and bioconjugation strategies for QD biosensors

Bioconjugation approaches encompass both covalent and non-covalent methodologies, each presenting distinct advantages and challenges that must be carefully evaluated for specific applications. Covalent bioconjugation strategies demonstrate superior stability with binding efficiencies reaching 86% for drug encapsulation and retention of 100% fluorescence properties over extended periods of 8 months, though these approaches often require complex multi-step synthesis procedures that can compromise QD optical properties (Fig. 6) [67, 68]. Non-covalent bioconjugation methodologies provide simplified preparation protocols while maintaining bioactivity, yet face challenges in achieving long-term stability and preventing ligand displacement in complex biological environments. Electrostatic interactions between QDs and biomolecules can achieve detection limits as low as 0.05 pg/mL for protein biomarkers, demonstrating the potential sensitivity of these approaches [69].

The integration of bioorthogonal chemistry approaches, particularly click chemistry methodologies, has emerged as a promising strategy to overcome traditional bioconjugation limitations while maintaining QD functionality. Fast binding reaction kinetics with response times less than 5 min enable rapid bioconjugation protocols that minimize QD exposure to potentially damaging reaction conditions [38]. However, the challenge of achieving site-specific conjugation without compromising QD stability remains significant, with many chemical approaches requiring optimization of reaction conditions to prevent QD aggregation or surface defect formation [70]. Zero-length coupling using EDC/NHS chemistry is the most widely adopted covalent conjugation strategy, demonstrating strong crosslinking capabilities and wide applicability across different biomolecule types, yet faces challenges in controlling reaction stoichiometry and preventing protein denaturation during the conjugation process [71]. The development of multivalent display systems on QD surfaces has shown promise for enhancing biological activity, with studies demonstrating 1.8-fold increases in aquaporin-4 expression and twofold improvements in water transport activity compared to monomeric controls, though achieving precise control over the number of biomolecules per QD remains a significant technical challenge requiring further development [72, 73].

### Non-specific binding minimization and biocompatibility enhancement

Polyethylene glycol (PEG) surface functionalization is employed to minimize non-specific protein adhesion and enhance the biocompatibility of QDs by the introduction of steric stabilization and hydrophilic surfaces. PEG coatings with molecular weights between 2000 and 5000 Da are documented as offering an optimal balance between minimal steric hindrance and improved protein resistance [74, 75].

Zwitterionic ligands, wherein positive and negative charges are present in the same molecule, exhibited lower protein adsorption and high colloidal stability over a range of pH values compared to PEG-functionalized surfaces. Sulfobetaine and carboxybetaine ligands have demonstrated very low protein adsorption in biological fluids while maintaining quantum yield, offering anti-fouling properties [76, 77].

Multidentate ligand systems improve colloidal stability via multiple coordination sites, which resist ligand displacement in complex biological environments. Polymer-based ligands with multiple thiol groups along the backbone have shown greater stability than small-molecule ligands, maintaining quantum yields above 70% even in buffers with physiological salt concentrations. Cross-linked polymer shells, produced via controlled radical polymerization, further stabilize QDs and can incorporate functional groups for bioconjugation, as well as properties that reduce immunogenicity [78–80].

**Fig. 7:**
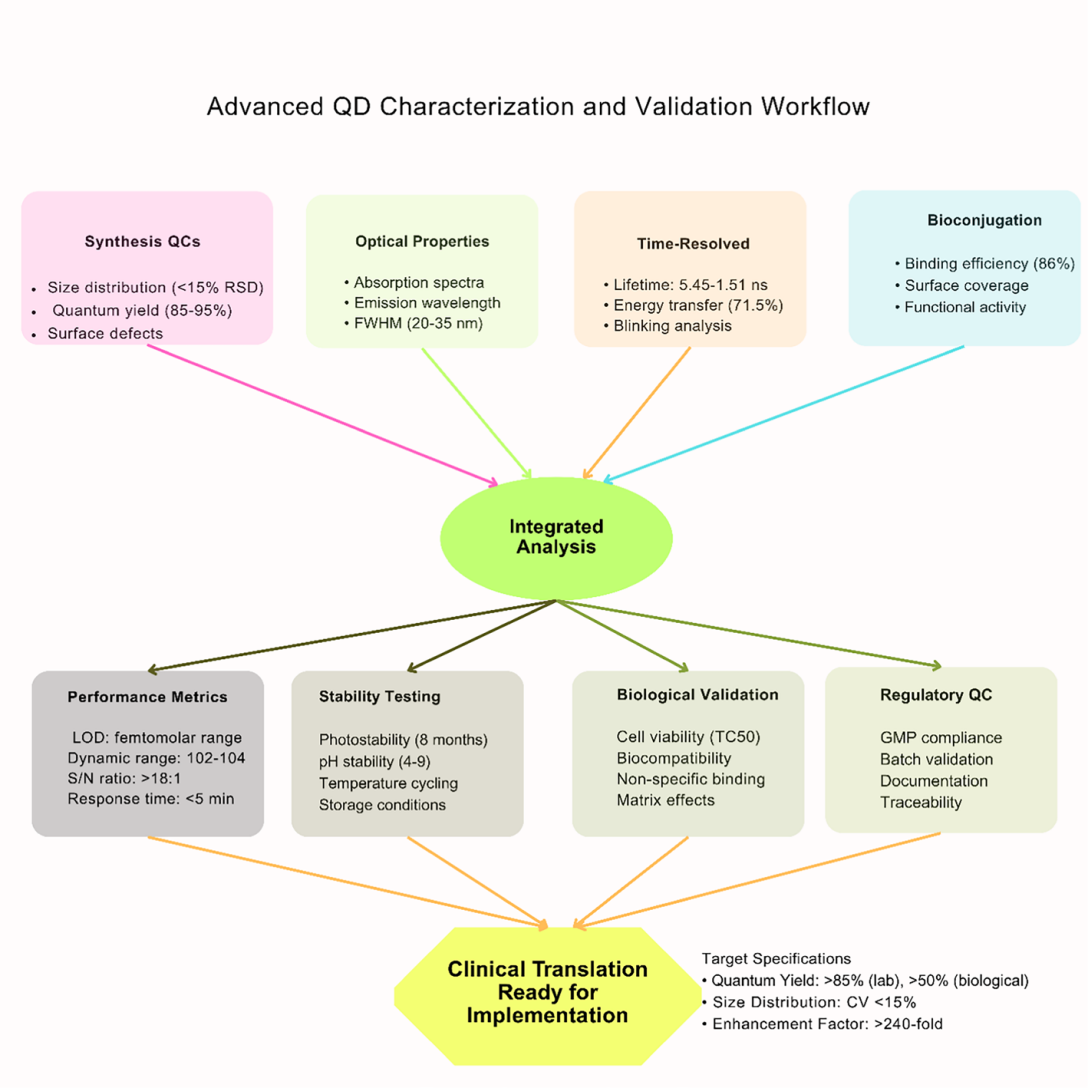
Schematic workflow for advanced characterization, validation, and clinical translation of QD biosensors

Surface engineering strategies have demonstrated significant effectiveness in reducing unwanted interactions, with PEGylation approaches showing particular promise for stabilizing QD suspensions and reducing non-specific binding to cells. [81]. Advanced protein-based stabilization methods using bovine serum albumin achieve superior properties compared to conventional approaches, with cross-linked systems demonstrating enhanced colloidal stability (Fig. 7) [82]. However, metal-containing QDs face toxicity concerns, limiting applications to in vitro bioanalysis, while carbon-based alternatives show promise for in vivo applications despite lower quantum yields [83, 84].

### Advanced characterization and validation protocols

Time-resolved measurements demonstrate energy transfer efficiencies reaching 71.5% with ultrafast lifetime variations from 5.45 to 1.51 ns [9]. Micropreparative gel electrophoresis enables purification with yields up to 90%, significantly outperforming conventional methods, though standardization challenges remain across different QD systems in complex biological matrices [84, 85].

## Photonic integration and signal enhancement technologies

**Fig. 8:**
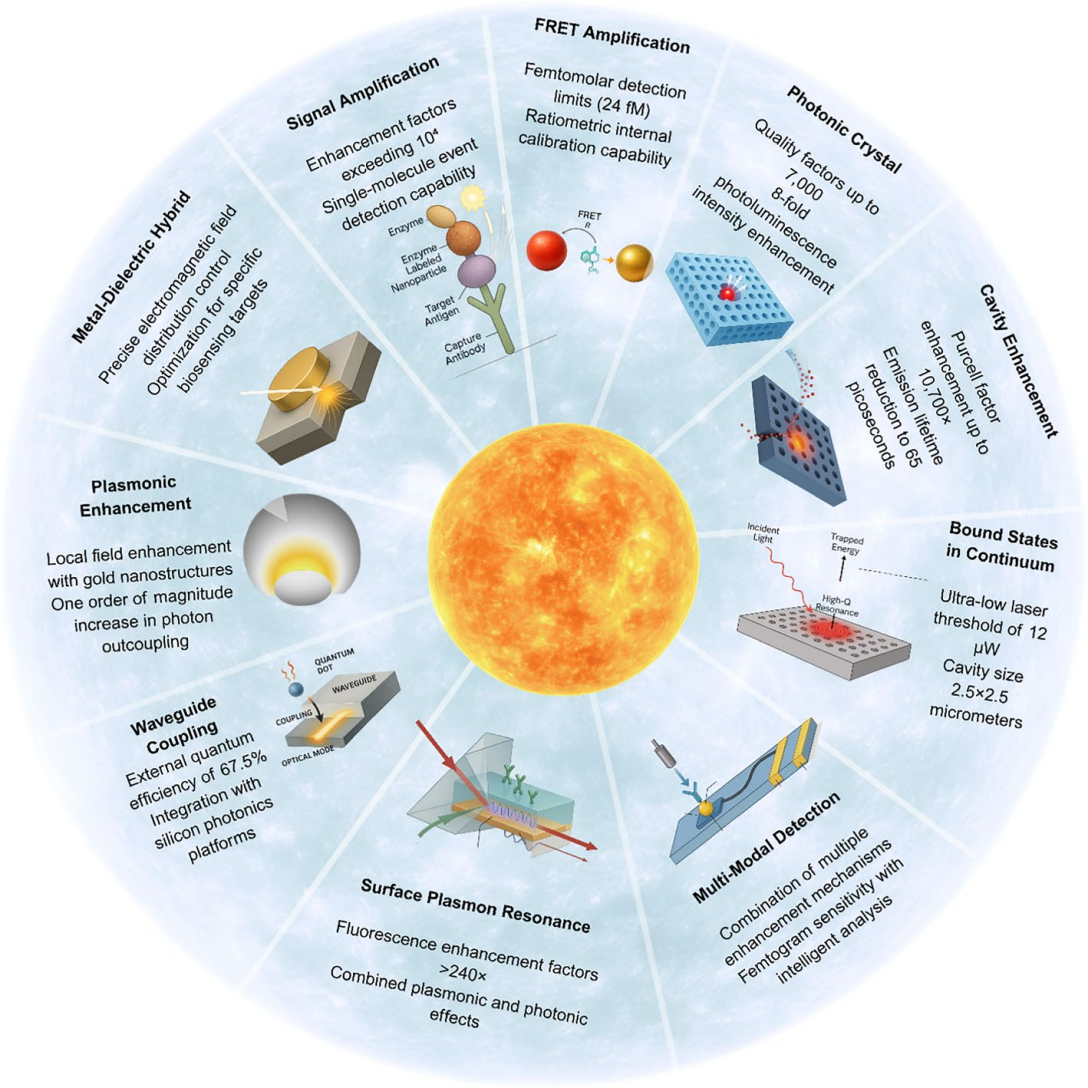
Brightness enhancement strategies for QD biosensors showing various signal amplification approaches and their corresponding enhancement factors for improved detection sensitivity

Integrating quantum dots with photonic structures can make their light emission much stronger and faster. In some advanced systems, this integration leads to Purcell factors over 10,700 and makes the quantum dots emit light in as little as 65 picoseconds. For comparison, traditional fluorescent labels usually have Purcell factors below 10 and emit light over several nanoseconds (Fig. 8) [86]. This strong enhancement means that quantum dot–photonic platforms can be much more sensitive than regular fluorescent biosensors. However, there are still some practical problems. Quantum dots can show signal instability over time because of blinking (turning on and off randomly) and photobleaching (gradual loss of brightness). During long measurements, these issues can cause the fluorescence signal to fluctuate by 30 to 70 percent, which makes it harder to get stable results [87]. Another challenge is making these biosensors on a large scale. Integrating quantum dots with photonic structures requires very precise and advanced manufacturing methods, like electron-beam lithography and exact alignment of optical parts. These steps make the production expensive and slow. Because of this, it is difficult to produce QD-photonic biosensors in large numbers for use in clinics or at the point of care [88, 89].

### Plasmonic enhancement and signal amplification mechanisms

Gold- boosted QD devices can increase light emission by over ten-fold when compared to unassisted devices, with high quantum efficiency being maintained. Conversely, unassisted systems radiate at five to ten-fold less efficiency [90]. Nevertheless, inherent losses associated with metallic components limit overall system efficiency, with quality factors typically 2–3 times lower than dielectric-only photonic structures [90].Strong local field enhancements can lead to unwanted heating effects and potential damage to biological samples under high excitation powers exceeding one mW/cm^2^.

### Photonic crystal cavities and bound state enhancement

**Fig. 9:**
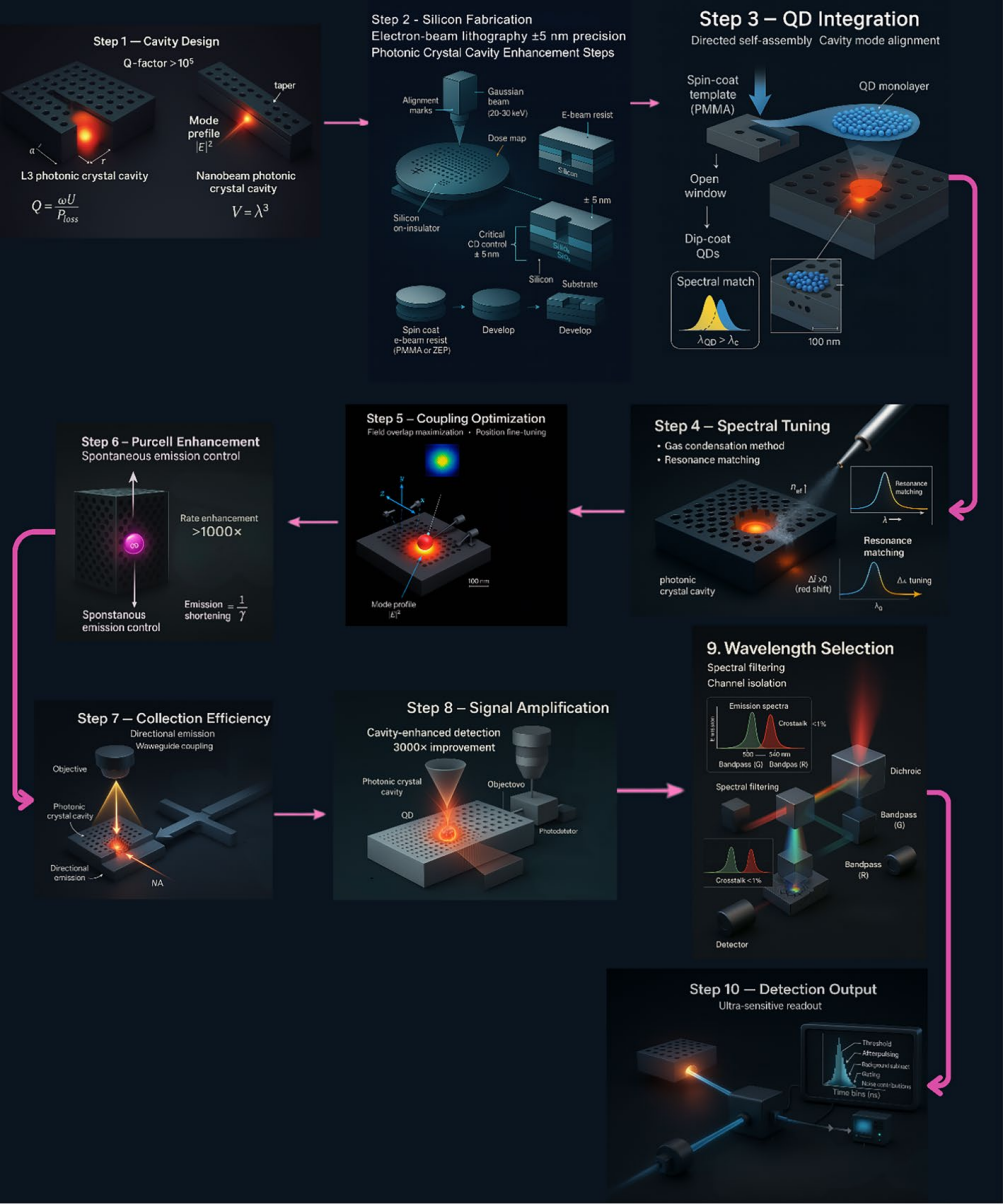
Photonic crystal cavity enhancement workflow for QD signal amplification

Continuum-bound states can reach quality factors up to 7000 and increase photoluminescence intensity by eight times compared to regular cavity systems, which usually have quality factors below 1000 (Fig. 9) [91]. However, creating these structures needs very precise and expensive nanoscale fabrication, and even small defects during production can reduce performance by 50 to 80 percent from the theoretical maximum. Also, these high-Q photonic crystal devices work well only over a narrow range of wavelengths, which means they are not ideal for biosensing applications that need to cover broad spectral ranges over 100 nm.

### Waveguide integration and on-chip detection

Heterogeneous integrated InAs QDs in silicon nitride waveguides achieve 67.5% external quantum efficiency at 1275 nm with linear photoresponse for input powers of 400 nW or less, in comparison with 20–30% efficiency for commercial silicon photodetectors [20].

### Fluorescence enhancement and signal amplification

Photonic structures can significantly increase the brightness of fluorescence signals. When plasmonic and photonic effects are combined, the signal can become more than 240 times stronger. This approach is more effective than using just one enhancement method, giving 10 to 20 times more signal boost. In these systems, plasmonic materials such as gold or silver focus light near the quantum dots, while photonic structures like photonic crystals help collect and amplify the emitted light [19]. Nonetheless, fluorescent-based systems commonly face issues like background emission from biological samples as well as photobleaching of the fluorescent labels with time. Such issues can compromise the signal-to-noise ratio by 2 to fivefold in the intricate biological environments. Even though it is feasible to enhance sensitivity through the combination of multiple signal enhancement strategies, it is crucial to control the spectral overlap as well as the interacting components’ distances carefully [92].

### Advanced detection integration

**Fig. 10:**
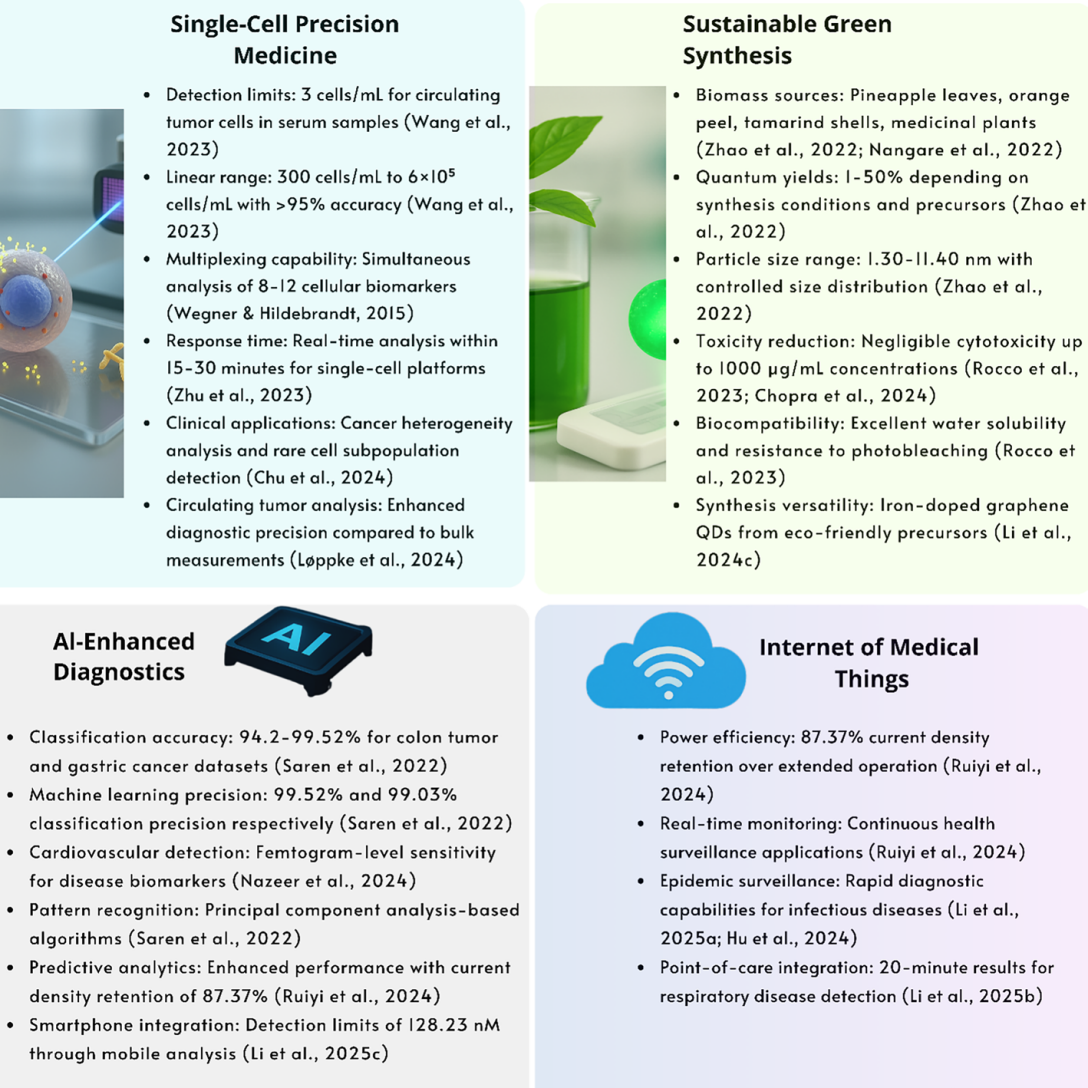
Future directions and emerging applications of QD biosensors

Traditional methods typically detect at the picogram scale, whereas machine learning-assisted photonic platforms have enhanced sensitivity, enabling the detection of cardiovascular biomarkers at femtogram levels (Fig. 10) [93]. Nevertheless, the utilization of these sophisticated systems presents data processing challenges that necessitate proficiency in algorithm development and photonics. For instance, dual-mode sensors that employ both fluorescence and colorimetric analysis can attain detection limits of 128.23 nM with smartphone-based readout [92].

## Detection mechanisms and analytical performance

Quantum dots are used as transducers in biosensing platforms and have enabled the precise and accurate Detection of biomolecules, diseases, and tiny compounds [94]. The detection mechanisms employed in QD-based biosensors are diverse and range from simple fluorescence quenching to complex multi-modal approaches that combine optical, electrochemical, and photoelectrochemical transduction methods [93, 95, 96].

### Fluorescence intensity-based detection

**Fig. 11:**
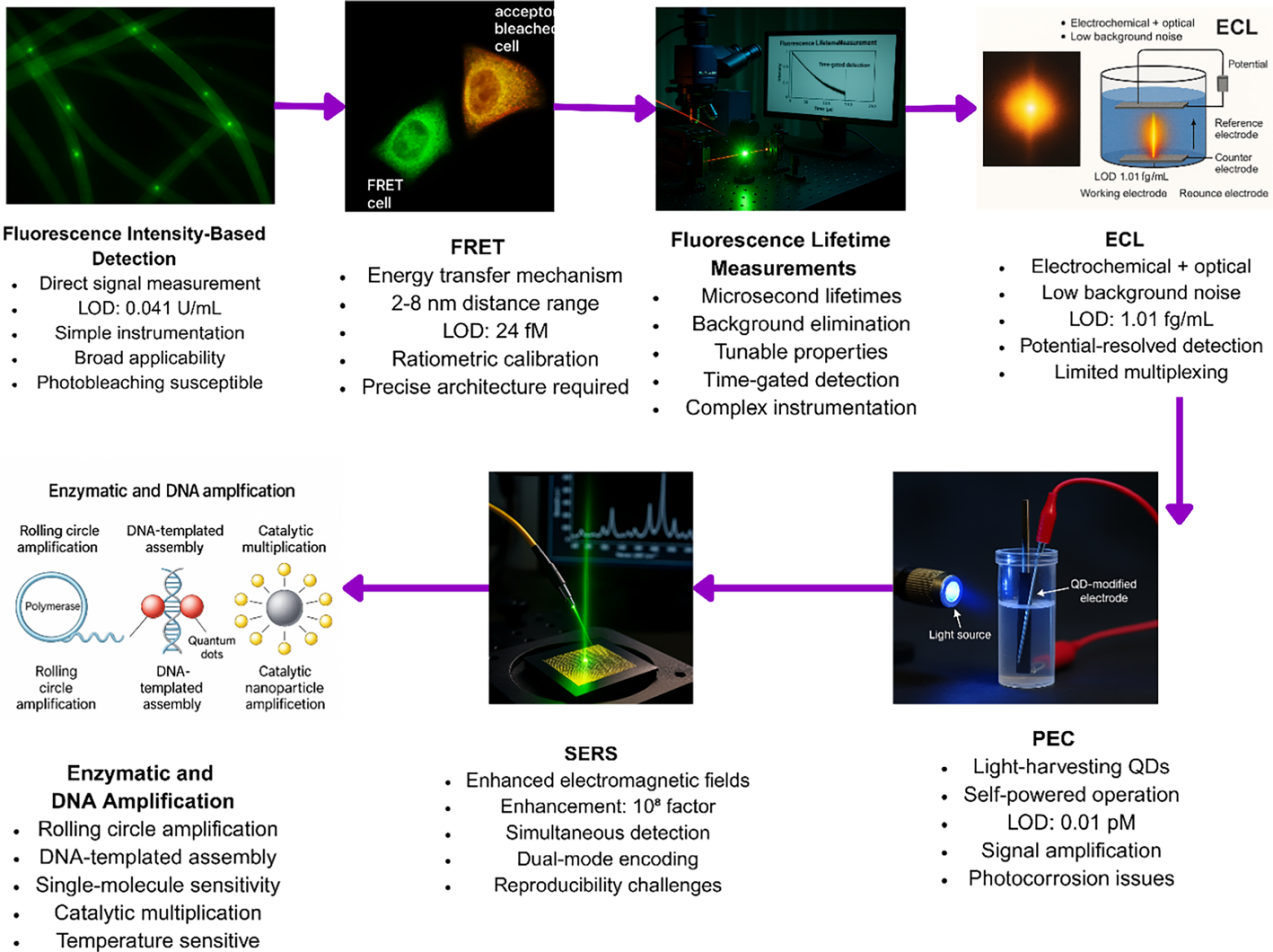
QD biosensor detection mechanisms, including fluorescence intensity, FRET, lifetime measurements, electrochemiluminescence, photoelectrochemical detection, and SERS, with representative experimental setups and performance metrics

Fluorescence intensity measurement remains the most widely used approach in QD-based biosensors (Fig. 11, Table 2) [97]. The emission wavelength of QDs can be precisely tuned by adjusting their size, enabling detection across the visible and near-infrared spectrum [94]. This property facilitates multiplexed assays where multiple targets can be detected simultaneously with a single excitation source. QD biosensors have demonstrated impressive analytical sensitivity across various applications. For example, silicon-doped carbon QDs achieved a detection limit of 0.041 U/mL for tyrosinase [2], and thiol-functionalized WS_2_ QDs enabled the detection of Staphylococcus aureus as low as 580 CFU/mL [98].

**Table 2:** Comparative performance analysis of QD biosensors versus conventional diagnostic platforms across key analytical parameters

Parameter	Biosensors	ELISA	PCR	Flow cytometry
Principle	Optical, electrical, or mechanical transducers; label-free or labeled	Enzyme-linked immunoassay	Nucleic acid amplification	Multiparametric fluorescence-based cell analysis
Target/analyte	Pathogen antigens, antibodies, nucleic acids, toxins, cells	Antigens, antibodies	DNA/RNA of pathogens	Cells and surface markers
Sensitivity (LOD)	Down to single pathogen or picogram levels (with amplification)	High (pg-ng/mL for proteins)	Extremely high (down to single copy)	High for cell populations
Dynamic range	Wide, especially with nanomaterials and amplification strategies	Moderate	Wide (several logs of nucleic acid)	Wide (multiple cell types/markers)
Specificity	High (with aptamers, antibodies, sequence-specific probes)	High, but sometimes cross-reactivity	Very high (primer/probe sequence)	Very high for surface markers
Multiplexing	High (chip-based or QD-based up to 10 + targets)	Low–moderate (1–3 targets/well)	Moderate (multiplex PCR possible)	Very high (> 10 markers/sample)
Time to result	15–30 min (sometimes < 15 min with microfluidics)	1–4 h (classic format)	1–3 h (including extraction/amplification)	1–2 h (sample prep + acquisition)
Ease of use	User-friendly, portable (esp. lateral flow), requires minimal training	Widely used, moderate complexity	Requires a skilled operator	Specialized training/equipment
Sample volume	Low (microliter scale)	Moderate (50–200 µL per well)	Low–moderate	Moderate to high (10^4–10^6 cells)
Clinical utility	Rapid POCT, outbreak response, decentralized labs	Clinical diagnostics, screening	Reference labs, high sensitivity	Immunophenotyping, cell analysis
Limitations	Matrix effects, integration challenges, validation needed	Labor intensive, cross-reactivity	Contamination risk, time-consuming	High instrument cost, complex

### Förster resonance energy transfer (FRET)

**Fig. 12:**
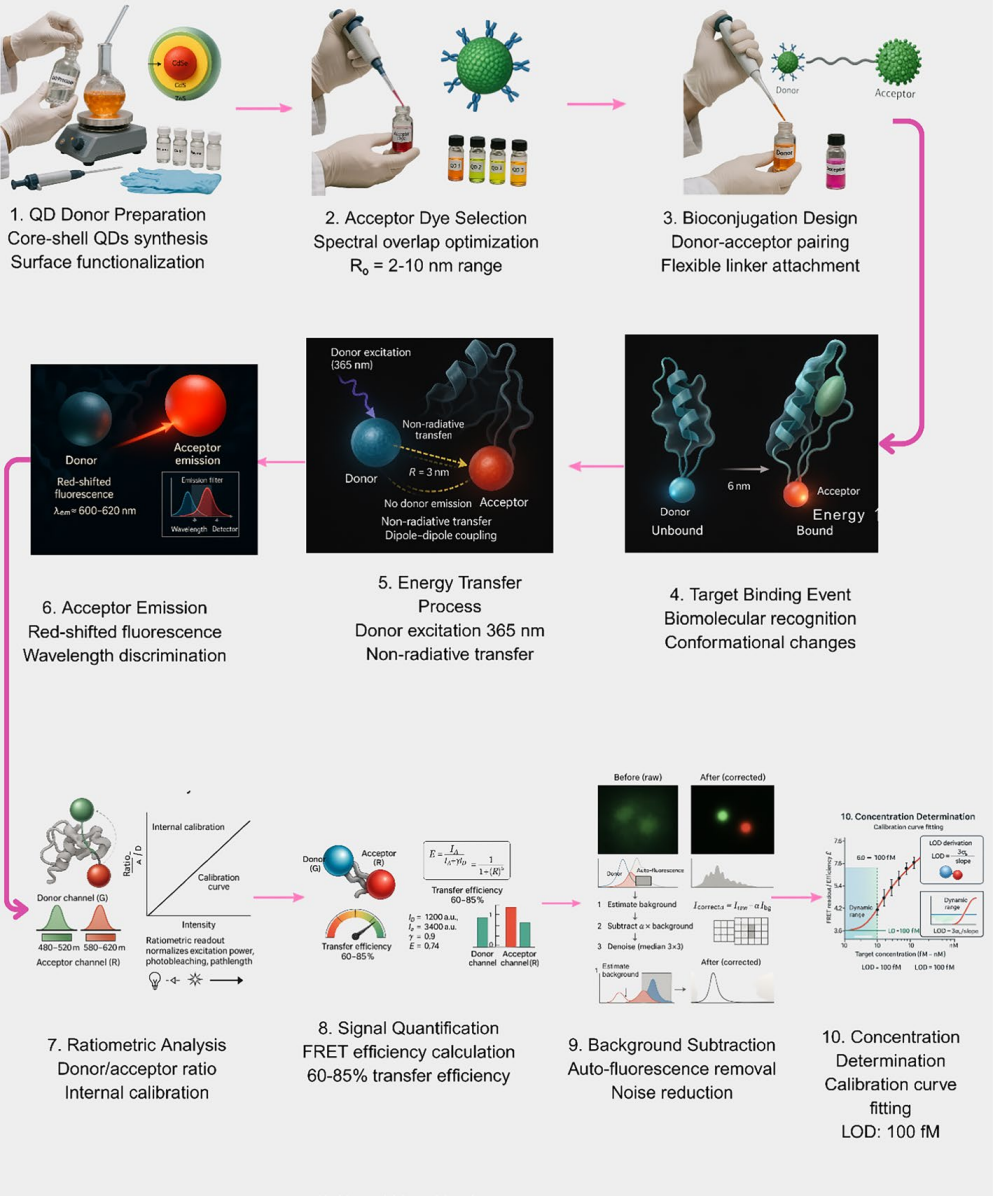
FRET-based QD detection mechanism demonstrating complete analytical workflow from probe preparation to quantitative analysis, achieving femtomolar sensitivity with ratiometric internal calibration

Förster Resonance Energy Transfer (FRET) applications using QDs as donors have achieved high performance due to their broad absorption spectra, narrow emission profiles, and large extinction coefficients, which provide efficient energy transfer to appropriately matched acceptor dyes or nanoparticles (Fig. 12, Table 3).

**Table 3:** Comparison of QD biosensor detection mechanisms and signal amplification strategies

Method	Key advantages	Limitations /challenges
Fluorescence intensity	Simple instrumentation & low cost Broad applicability to many analytes	Photobleaching leads to signal loss Matrix interference & signal drift
FRET (Förster Resonance Energy Transfer)	Ultralow LOD (~ 24 fM) Ratiometric self-calibration Very low background	Efficiency limited to 2–8 nm donor–acceptor distance Requires precise molecular architecture Environmental sensitivity (pH, temperature)
Fluorescence lifetime	Time-gated detection removes background Lifetime tunable via QD design Discriminates from autofluorescence	Complex, expensive TCSPC/laser setup Narrower dynamic range Higher operational cost
Electrochemiluminescence (ECL)	Extremely low background noise LOD Precise electrochemical control	Electrode fouling in biological media Limited multiplexing Needs co-reactant (e.g., TPrA, persulfate)
Photoelectrochemical (PEC)	Self-powered or low-power operation Intrinsic signal amplification Femtomolar sensitivity	QD photocorrosion under light Charge recombination losses Strict light-intensity/wavelength control
Surface-enhanced raman scattering (SERS)	Enhancement factors up to 10^8^ Simultaneous (multiplex) detection Dual optical/electro “barcode” options	Hot-spot positioning Reproducibility issues across substrates Complex, costly substrate fabrication
Enzymatic & DNA amplification (e.g., RCA, HCR)	Single-molecule sensitivity Massive signal multiplication High sequence specificity	Temperature-sensitive enzymes Multi-step, time-intensive protocols Risk of non-specific amplification

Advanced FRET architectures enable simultaneous monitoring of multiple enzymatic activities. They also monitor protein–protein interactions. [99] constructed a fluorescence sensing platform for Golgi protein 73 detection based on FRET between nitrogen-doped graphene QDs and molybdenum disulfide nanosheets, achieving linear detection in the range of 5–100 ng/mL with a detection limit of 4.54 ng/mL [99]. Ratiometric measurements provide internal calibration and improve quantitative accuracy with recovery rates of 97.21–100.83% in human serum samples [99, 100].

### Fluorescence lifetime measurements

Fluorescence lifetime measurements exploit the microsecond-scale emission lifetimes characteristic of QDs compared to nanosecond lifetimes of conventional fluorophores and biological autofluorescence, enabling time-gated detection schemes that effectively eliminate background interference. QD lifetimes can be systematically tuned through modifications in composition, size, and surface chemistry, achieving enhanced sensitivity for specific applications [101].

### Hybrid and multimodal detection approaches

#### Electrochemiluminescence (ECL) detection

Electrochemiluminescence (ECL) detection combines the precision of electrochemical control with the sensitivity of optical readout, resulting in low background noise and detection limits. [102]. Innovations in ECL biosensors have capitalized on this synergy to achieve ultra-sensitive analyses for a variety of clinically important biomarkers. For example, [103] developed a potential-resolved differential ECL immunosensor by integrating MOF-5-wrapped CdS QDs, enabling simultaneous detection at two distinct potentials and achieving an impressive detection limit of 5.01 fg/mL for cardiac troponin I [103]. Similarly, Zhao et al. reported a high-performance signal-on/off ECL gel aptasensor using a controlled release mechanism for prostate-specific antigen, attaining detection limits as low as 1.01 fg/mL [14]. These advances demonstrate how the combination of QDs with metal–organic frameworks and signal amplification strategies can push ECL biosensing into the femtomolar range [104, 105].

Despite these achievements, several technical and practical challenges remain for widespread ECL biosensor adoption. Electrode fouling by complex biological matrices continues to undermine long-term signal stability and reproducibility [103]. The necessity for co-reactants such as tripropylamine or persulfate not only complicates assay protocols but can also introduce new sources of interference [104]. Moreover, while ECL offers high sensitivity, its multiplexing capacity still lags behind purely optical detection methods, limiting simultaneous multi-analyte measurements. The inherent complexity of ECL mechanisms, often involving multiple competing electrochemical and luminescent pathways, can make signal interpretation challenging, especially in real biological samples [102]. Furthermore, interference from endogenous redox-active species poses additional hurdles to selectivity and accuracy.

#### Photoelectrochemical (PEC) detection

Photoelectrochemical (PEC) sensors leverage QDs as highly efficient light-harvesting sensitizers, generating measurable photocurrents upon illumination [106, 107]. In these systems, specific target binding events alter the efficiency of charge transfer at the electrode interface, producing detectable changes in current and enabling sensitive quantification of biomolecules [108]. For instance, Ouyang et al. developed a self-powered PEC aptasensor based on Au/CeO_2_/g-C_3_N_4_ heterostructures for the ultrasensitive detection of microcystin-LR, achieving a detection limit of 0.01 pM without the need for external power sources [106]. Through signal amplification mechanisms intrinsic to the PEC process, these platforms routinely reach detection limits in the femtomolar range for both DNA and protein biomarkers [109]. Huang et al. for example, designed a dual-engine-powered paper PEC platform incorporating a 3D DNA nanomachine-mediated CRISPR/Cas12a system, allowing simultaneous detection of multiple miRNAs with detection limits of 5.5 fM for miRNA-141 and 3.4 fM for miRNA-21 [107, 109, 110].

Despite these advances, PEC detection systems still face significant operational and technical challenges. Photocorrosion of QD sensitizers under prolonged illumination can cause signal degradation and limit sensor lifespan. The need for simultaneous optical and electrochemical measurement increases the overall complexity and cost of instrumentation [109]. Charge recombination phenomena may further reduce photoconversion efficiency, thereby limiting ultimate sensitivity. Additionally, dissolved oxygen and other electroactive species present in biological samples can create competing redox reactions, compromising signal reliability. Achieving reproducible results also requires precise control over illumination intensity and wavelength, adding to the experimental challenges [106].

#### Surface-enhanced raman scattering (SERS)

The integration of QDs with plasmonic nanostructures in SERS platforms dramatically amplifies both photoluminescence and Raman scattering from target molecules, enabling ultrasensitive detection. For instance, [11] achieved enhancement factors up to 10^8^ using QD-encapsulated nanogels for virus detection [111].

Despite these advances, SERS-based biosensors face practical challenges: reproducibility is limited by the need for precise analyte placement in electromagnetic hot spots, and heterogeneous substrate enhancement complicates quantitative analysis [112]. Competition between QD fluorescence and Raman signals, spectral interference from biological samples, and the complexity of fabricating SERS-active substrates also constrain real-world applications [112].

#### Signal amplification strategies

Such as enzymatic amplification, rolling circle amplification, and catalytic nanoparticle systems, enable single-molecule sensitivity by producing thousands of detectable events from a single binding event [113]. For example, [113] achieved femtomolar detection limits for multiplexed microRNA analysis using QD-based digital particle counting [113]. DNA-templated assembly further allows QDs to form distinct optical patterns for digital molecular counting with high specificity. [103] also reported an ECL aptasensor for Pb^2^⁺ detection with a detection limit as low as 0.19 fM using dual amplification strategies [104].

However, these approaches face limitations: enzymatic systems can be inhibited by sample components, and multi-step amplification increases assay complexity and risk of false positives. [113]. Temperature sensitivity and longer assay times also affect practicality and cost-effectiveness. [109].

## Single-cell analysis

**Fig. 13:**
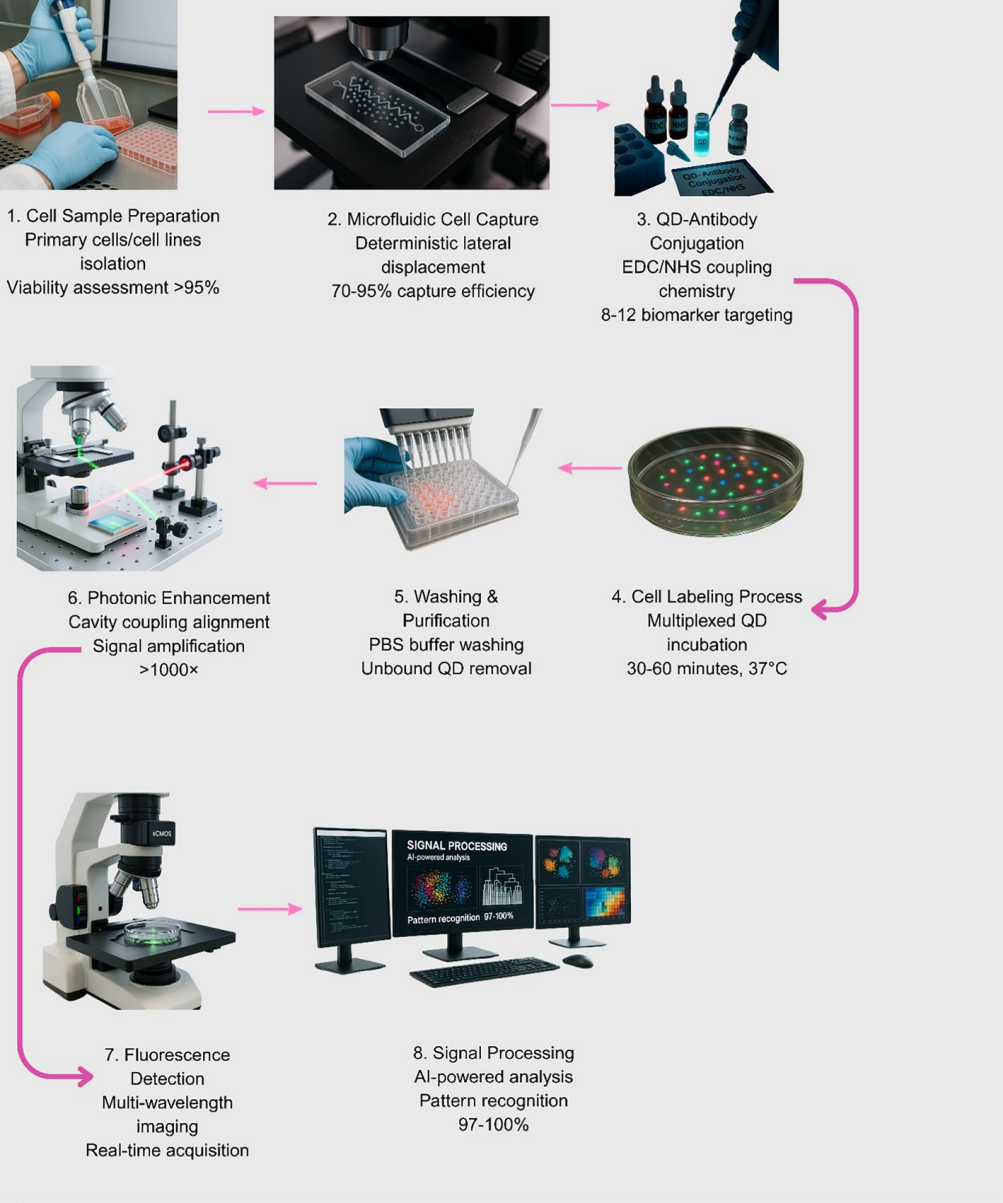
Single-cell QD biosensing platform workflow from cell isolation to clinical decision-making, enabling precision medicine applications with > 1000 × signal amplification and 97–100% accuracy for biomarker quantification

Single-cell analysis enables the detection of rare cell subpopulations, providing higher diagnostic precision compared to bulk measurements (Fig. 13) [114]. QD-enhanced single-cell biosensors leverage unique photophysical properties, including broad absorption spectra, narrow emission linewidths, and exceptional photostability, to enable simultaneous detection of multiple biomarkers within individual cells with unprecedented sensitivity. These platforms demonstrate remarkable capabilities in identifying circulating tumor cells at concentrations as low as 1–10 cells per milliliter, detecting rare immune cell subpopulations comprising less than 0.1% of total populations, and monitoring dynamic protein expression changes within single cells [5]. The multiplexing capabilities afforded by size-tunable QD emission enable simultaneous tracking of 8–12 distinct cellular markers using single excitation sources, providing comprehensive phenotypic characterization essential for understanding disease progression and therapeutic response [115]. Integration with microfluidic platforms enables real-time monitoring of cellular drug responses, identification of therapy-resistant populations, and characterization of cell-to-cell communication mechanisms critical for personalized therapeutic strategies [96].

### Cancer diagnostics and circulating tumor cell detection

Single-cell analysis of cancer cells has improved our understanding of tumor heterogeneity and metastatic processes. Advances in single-cell RNA sequencing (scRNA-seq) and computational analysis have enabled characterization of cellular diversity within tumor microenvironments, revealing previously hidden cellular subpopulations that play roles in disease progression [96]. Circulating tumor cells (CTCs) represent a particularly valuable target for QD-based single-cell analysis due to their rarity and clinical significance. These cells, present at frequencies as low as 1–100 cells per billion blood cells, require highly sensitive detection methods that can distinguish them from normal circulating cells (Table 2) [116].

### Cardiovascular disease biomarkers and QD applications

QD-based platforms have demonstrated promising potential for diagnosing and monitoring cardiovascular disease by detecting key cardiac biomarkers. Functionalized graphene QDs have achieved sensitive detection of cardiac troponin I with limits as low as 0.02 ng/mL using antibody-free electrochemical approaches, while maintaining excellent stability and biocompatibility [117]. Silicon QD theranostics integrated with network analysis of metabolomic data have identified biomarkers, including CD44 and myristic acid, for cardiac ischemia applications [118]. Furthermore, targeted drug delivery systems utilizing curcumin-loaded PEGylated graphene QDs have shown promising results in myocardial infarction treatment, with demonstrated improvements in cardiac function and reduced oxidative stress [119]. Angiotensin 1 peptide-conjugated CdSe/ZnS QDs have enabled cardiac-specific delivery for hydrogen sulfide therapy in ischemia–reperfusion injury, achieving enhanced local therapeutic effects while minimizing systemic side effects [120]. The heterogeneity of single-cell responses and the potential for QD-induced cellular perturbations during measurement raise questions about the accuracy of single-cell QD-based analyses, particularly when extrapolating results to broader cellular populations [114].

### Infectious disease detection and QD applications

QD-based platforms are good tools for infectious disease detection, offering sensitivity and rapid diagnostic capabilities across diverse pathogenic targets. Fluorescent QD-based biosensors have demonstrated performance in tuberculosis detection, achieving detection limits as low as 0.13 amol/L using dual signal amplification strategies combining recombinase polymerase amplification with catalytic hairpin assembly [121]. These systems successfully identified tuberculosis in clinical sputum samples from 36 patients with results highly consistent with real-time quantitative PCR, establishing their clinical viability. For SARS-CoV-2 detection, QD-enhanced immunochromatographic assays have achieved sensitivity improvements, with detection limits of 1.427 pg/mL for nucleocapsid protein and positive detection rates increasing from 55.17 to 86.67% compared to commercial products through multiple aptamer recognition strategies [122]. Hepatitis detection applications have utilized innovative approaches including MXene QDs in bipolar electrochemiluminescent platforms, achieving detection limits of 3.3 × 10⁻^5^ ng/mL for hepatitis C virus envelope protein E2 through photothermal amplification mechanisms [123]. HIV detection has been changed by graphene QD-based electrochemical sensors, achieving femtogram-level sensitivity for HIV RNA detection, with the sensor maintaining response for 60 days at room temperature while discriminating between HIV-negative and positive samples with high specificity and stability. Carbon QDs have shown broad-spectrum antimicrobial activity, with multifunctional platforms incorporating nitrogen, sulfur, and copper doping achieving bacterial infection treatment through mechanisms including inflammation control, bacterial inhibition, immunomodulation, and angiogenesis promotion [124].

### Periodontal disease biomarkers and QD detection

Periodontal disease diagnosis has been transformed through QD-based detection of key inflammatory biomarkers, including matrix metalloproteinases-8 (MMP-8), interleukin-1β (IL-1β), and tumor necrosis factor-alpha (TNF-α) [125]. QD immunoassays demonstrate superior performance with detection limits of 1–5 ng/mL for MMP-8 and 1–100 pg/mL for inflammatory cytokines through multiplexed detection capabilities [42]. Clinical validation studies show effective differentiation between periodontal patients and healthy individuals with signal-to-noise ratios exceeding 18:1, while emerging microRNA detection platforms achieve femtomolar sensitivity for early disease detection before clinical manifestation [125].

## Clinical translation

QD biosensor commercialization faces economic barriers, with manufacturing costs representing the primary obstacle to market penetration due to complex fabrication techniques [89]. Advanced synthesis methods achieve relative standard deviations below 15% for particle size distribution and coefficients of variation under 10% for diagnostic parameters [126]. QD-based assays typically provide dynamic ranges of 10^2^–10^4^, which may be insufficient for clinical applications requiring detection across 5–6 orders of magnitude [79, 127–129].

Clinical validation studies demonstrate promising results, with QD biosensors achieving 92.3–98.1% favorable rates compared to 78.3–83.1% for traditional ELISA in COVID-19 detection, with response times reduced to 5 min [130]. Detection capabilities range from femtomolar (10⁻^15^ M) concentrations for high-affinity interactions to 1–100 pM for standard biomolecular assays [122, 127, 131, 132].

### Regulatory compliance and safety

QD systems demonstrate tenfold improved sensitivity for rare cell detection and threefold enhanced sensitivity compared to conventional methods, with 50% reduction in analysis time, positioning them as next-generation diagnostic tools [115]. Signal enhancement factors can exceed 10^4^ under optimized conditions, with multiplexing capabilities enabling 8–12 targets to be simultaneously detected using single excitation sources. [133].

QD biosensors have demonstrated significant clinical potential across multiple diagnostic applications, with several platforms achieving sensitivity levels comparable to or exceeding conventional laboratory methods.

Clinical validation studies have extended to multiple disease areas, demonstrating the versatility of QD biosensor platforms. A QD nanobead-monoclonal antibody probe-based immunochromatographic assay for Trichinella spiralis infection monitoring achieved 100% selectivity compared to 90% for commercial ELISA kits, with response times reduced to 25 min [23].

### Point-of-care implementation and clinical workflow integration

Integrating QD biosensors into clinical workflows requires transitioning from laboratory-based to point-of-care diagnostics. Multiplex fluorescence lateral flow immunoassays utilizing QD nanobeads have achieved simultaneous detection of SARS-CoV-2, adenovirus, and influenza A virus with detection limits of 56, 120, and 41 copies/mL, respectively, providing results within 20 min [24]. These platforms demonstrate LOD improvements of 200–1220 times compared to colloidal gold lateral flow assays while maintaining sensitivity comparable to PCR techniques.

Clinical feasibility has been further demonstrated through successful implementation in complex biological matrices. A dual-mode lateral flow biosensor for gastric cancer markers achieved ultra-low detection limits of 6.9 pM for pepsinogen I and 15.7 pM for pepsinogen II, with good linear correlation to commercial time-resolved fluoroimmunoassays in clinical sample analysis [134].

### Regulatory compliance and quality assurance

**Fig. 14:**
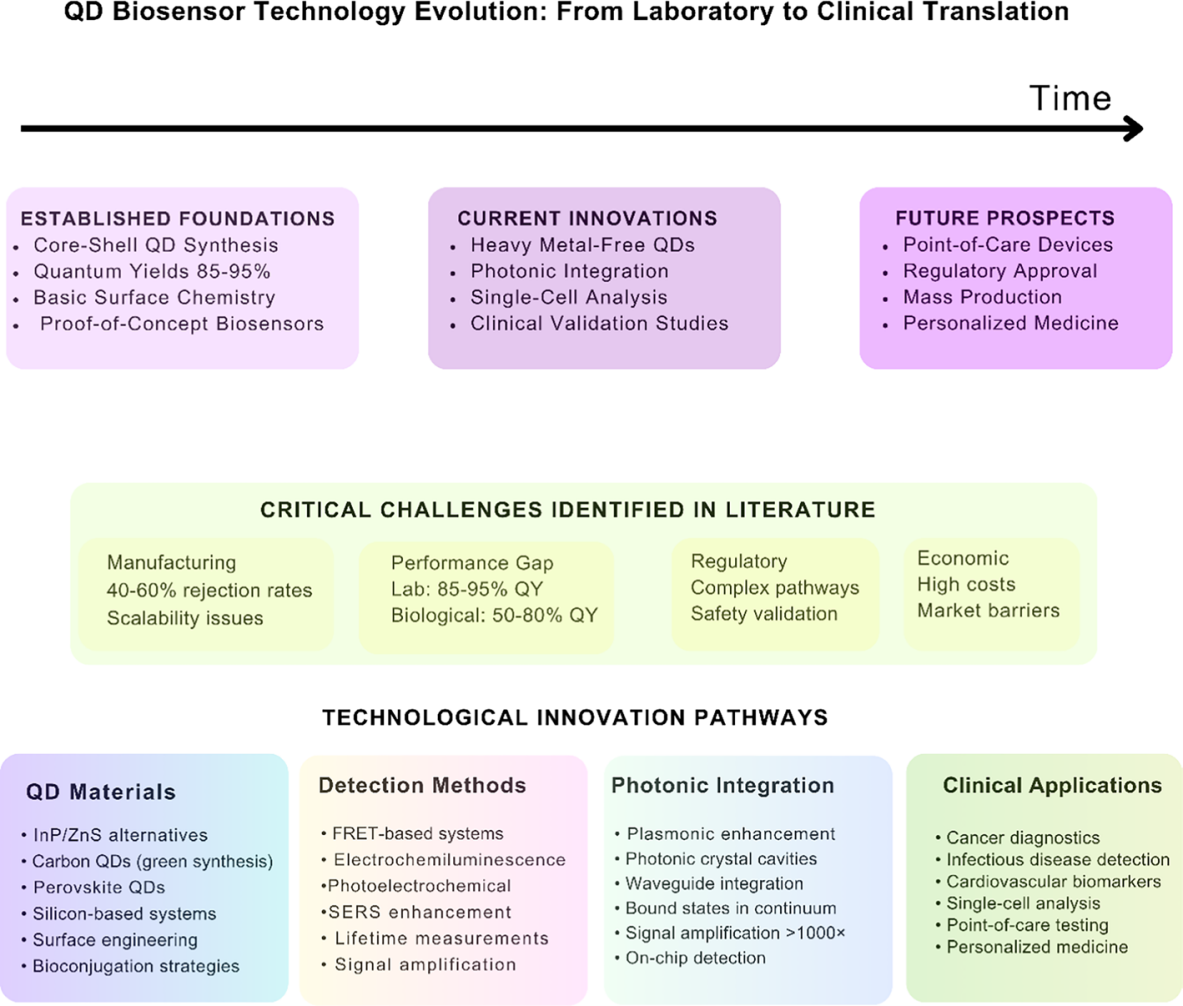
Evolution and future directions of quantum dot-enhanced biosensor technology. The timeline illustrates the progression from established laboratory foundations to current innovations and future clinical translation prospects

The regulatory pathway for QD biosensors requires validation protocols addressing both analytical and clinical performance standards. Biosensors targeting specific biomarkers such as miR-122 for breast cancer detection have demonstrated clinical validation with 100% sensitivity and 100% specificity, achieving detection limits of 0.8 zM across linear ranges from 0.001 aM to 1000 nM (Fig. 14) [135].

Quality control measures have been implemented across multiple platforms to ensure reproducibility and reliability in clinical settings. A biosensor for procalcitonin detection utilizing QD-enhanced electrochemiluminescence achieved a detection limit of 0.029 pg mL⁻^1^ with good selectivity and stability, demonstrating successful application in actual patient samples [132, 136–139].

Principal component analysis-based machine learning algorithms combined with multicolor QD immunobiosensors have achieved classification precision of 99.52% and 99.03% for colon tumor and gastric cancer datasets, respectively, with classification accuracy of 94.86% and 94.2% [140].

Fluorescence-based immunochromatographic assays for fetuin-B detection have achieved detection limits of 0.299 ng mL^−1^ with broad linear ranges and good correlation with commercial ELISA kits (r = 0.98451, n = 116), demonstrating commercial viability for cardiovascular disease screening applications [89, 141, 142].

Cytotoxicity evaluations have demonstrated that QD toxicity is highly dependent on surface modifications, particle size, and exposure conditions [143]. Studies utilized OPA-modified CdSe/ZnS core/shell QDs in human skin cells. TC50 values ranged from 55.0 to 102.1 nM in malignant melanoma cells. HaCaT keratinocyte cells demonstrated significantly lower toxicity with TC50 values of 162.0 to 818.2 nM [143]. These findings highlight the importance of cell-type-specific toxicity assessment and the potential for targeted therapeutic applications.

Mitigation strategies for QD cytotoxicity have shown results through various approaches. Pretreatment with natural antioxidants such as silibinin significantly reduced QD-induced cell death, suggesting that antioxidant supplementation can modulate QD-induced cytotoxicity through membrane stabilization and oxidative stress reduction [143]. Microencapsulation is another effective strategy for toxicity mitigation, where CdSe/ZnS-polyethyleneimine QDs encapsulated in polymeric microcapsules terminated with polyethylene glycol effectively protected human fibroblasts from acute cytotoxic effects while maintaining particle luminescence for sensing applications [31, 144, 145].

### Regulatory framework and clinical safety

Genetic circuits designed to detect nanomaterial-triggered toxicity through engineered heat shock response mechanisms have provided rapid toxicity assessment capabilities [146]. Clinical translation of QD biosensors requires adherence to stringent safety standards established by regulatory agencies, including the FDA and EMA [147].

### Quality parameters and manufacturing standards

Relative standard deviation requirements are typically below 15% for particle size distribution. Coefficients of variation must be under 10% for key diagnostic parameters [126]. Essential quality specifications encompass photostability testing under accelerated aging conditions, assessment of bioconjugation efficiency, optimization of antibody-to-QD ratios, and evaluation of binding kinetics in complex biological matrices [148]. Cost-effective and cell-based bioassay methods are essential tools for evaluating biosensor performance, providing standardized approaches for bioactivity assessment that complement traditional analytical characterization techniques [149–156].

### Future directions and implementation challenges

Standardization efforts must balance the need for high-quality control with cost-effectiveness. Integration of artificial intelligence and machine learning approaches into quality control systems offers potential for improved process monitoring and predictive quality assessment, though implementation requires careful validation and regulatory approval [157].

The development of user-friendly detection platforms that maintain high analytical performance while reducing complexity is a goal for future improvements [154]. Despite significant advances, including quantum efficiencies approaching 98% and signal amplification of more than 1000-fold by integration into advanced photonic structures, the significant reduction in efficiency in biological environments and the need for standardized protocols for accurate evaluation of clinical performance remain major obstacles to commercialization. In addition, moving towards more sustainable synthesis methods, such as heavy metal-free quantum dots, is essential for achieving broader clinical applications and reduced toxicity [158, 159].

## Conclusion

QD-enhanced photonic biosensors have demonstrated quantum efficiencies up to 98 percent and signal amplification factors greater than one thousand under optimized laboratory conditions. The introduction of heavy metal-free alternatives such as indium phosphide and carbon-based QDs has addressed safety concerns while still enabling detection limits in the femtomolar range for high-affinity biomarkers. Clinical validation studies show promising sensitivity; for example, COVID-19 antibody assays have achieved sensitivities between 92.3 and 98.1 percent, which is higher than conventional ELISA, and circulating tumor cell detection has reached limits as low as three cells per milliliter. Manufacturing scalability remains challenging. Rejection rates reach 40–60% in pharmaceutical-grade production. Quantum yield often declines from 85–95% in laboratory settings to 50–80% in biological samples. Regulatory approval for diagnostics based on nanotechnology is still complex, and high production costs present further obstacles to commercialization. Bridging the gap between laboratory success and clinical application will require thorough validation of long-term stability, standardization of regulatory pathways, and the development of scalable and cost-effective manufacturing processes.

## Data Availability

No datasets were generated or analysed during the current study.
